# Potent inhibition of rhabdoid tumor cells by combination of flavopiridol and 4OH-tamoxifen

**DOI:** 10.1186/1471-2407-10-634

**Published:** 2010-11-19

**Authors:** Velasco Cimica, Melissa E Smith, Zhikai Zhang, Deepti Mathur, Sridhar Mani, Ganjam V Kalpana

**Affiliations:** 1 Department of Genetics, Albert Einstein College of Medicine, 1300 Morris Park Avenue, Bronx, New York 10461 USA; 2 Department of Medicine, Albert Einstein College of Medicine, 1300 Morris Park Avenue, Bronx, New York 10461 USA; 3 Albert Einstein Cancer Center, Albert Einstein College of Medicine, 1300 Morris Park Avenue, Bronx, New York 10461 USA

## Abstract

**Background:**

Rhabdoid Tumors (RTs) are highly aggressive pediatric malignancies with poor prognosis. There are currently no standard or effective treatments for RTs in part because treatments are not designed to specifically target these tumors. Our previous studies indicated that targeting the cyclin/cdk pathway is a novel therapeutic strategy for RTs and that a pan-cdk inhibitor, flavopiridol, inhibits RT growth. Since the toxicities and narrow window of activity associated with flavopiridol may limit its clinical use, we tested the effect of combining flavopiridol with 4-hydroxy-Tamoxifen (4OH-Tam) in order to reduce the concentration of flavopiridol needed for inhibition of RTs.

**Methods:**

The effects of flavopiridol, 4OH-Tam, and their combination on RT cell cycle regulation and apoptosis were assessed by: i) cell survival assays, ii) FACS analysis, iii) caspase activity assays, and iv) immunoblot analysis. Furthermore, the role of p53 in flavopiridol- and 4OH-Tam-mediated induction of cell cycle arrest and apoptosis was characterized using RNA interference (siRNA) analysis. The effect of p53 on flavopiridol-mediated induction of caspases 2, 3, 8 and 9 was also determined.

**Results:**

We found that the combination of flavopiridol and 4OH-Tam potently inhibited the growth of RT cells. Low nanomolar concentrations of flavopiridol induced G_2 _arrest, which was correlated to down-modulation of cyclin B1 and up-regulation of p53. Addition of 4OH-Tam did not affect flavopiridol-mediated G_2 _arrest, but enhanced caspase 3,7-mediated apoptosis induced by the drug. Abrogation of p53 by siRNA abolished flavopiridol-induced G_2 _arrest, but enhanced flavopiridol- (but not 4OH-Tam-) mediated apoptosis, by enhancing caspase 2 and 3 activities.

**Conclusions:**

Combining flavopiridol with 4OH-Tam potently inhibited the growth of RT cells by increasing the ability of either drug alone to induce caspases 2 and 3 thereby causing apoptosis. The potency of flavopiridol was enhanced by abrogation of p53. Our results warrant further studies investigating the combinatorial effects of flavopiridol and 4OH-Tam as a novel therapeutic strategy for RTs and other tumors that have been shown to respond to flavopiridol.

## Background

RTs, including Malignant Rhabdoid Tumors (MRT), Atypical Teratoid and Rhabdoid Tumors (AT/RT), and extra renal rhabdoid tumors (ERRT) are rare, but highly aggressive pediatric solid tumors with poor prognosis [1]. Current therapy for RTs includes surgical resection, radiation therapy, and/or chemotherapy with empirically selected and highly toxic chemotherapeutics, which are largely ineffective [2,3]. Despite aggressive treatment, mean survival with surgical intervention alone is only 3 months and with adjuvant chemotherapy and radiotherapy is only 8 months [4]. Therefore, strategies based on understanding the genesis of RTs will aid in the development of novel therapies. RTs are characterized by biallelic deletions and/or mutations in *INI1/hSNF5*, a tumor suppressor and component of the chromatin remodeling SWI/SNF complex [5,6]. Reintroduction of INI1/hSNF5 into RT cells induces G_1 _cell cycle arrest and senescence. INI1/hSNF5 mediates these effects by directly activating p16^Ink4a ^by recruiting the SWI/SNF complex and by directly repressing *cyclin D1 *by recruiting the HDAC1 complex [7-10]. We have found that *cyclin D1 *is de-repressed in human and mouse RTs and is required for rhabdoid tumorigenesis in mouse models [9,11,12]. Such studies indicated that therapeutic targeting of cyclin D1 and its pathway could be an effective and novel therapeutic strategy for RTs.

We previously reported that down-modulating cyclin D1 and inhibiting cyclin dependent kinases (cdks) using either flavopiridol or a combination of *N*-(4-hydroxyphenyl)retinamide (4-HPR) with 4OH-Tam is effective in inhibiting RTs *in vitro *and in xenograft tumor models *in vivo *[11,13]. The effectiveness of 4-HPR and flavopiridol was correlated with down-modulation of cyclin D1 in xenograft tumors [13].

Flavopiridol is one of the first cdk inhibitors to enter clinical trials. Although early clinical trials were unsuccessful, design of a novel schedule of administration based on the *in vitro *and *in vivo *pharmacokinetic modeling of flavopiridol's effect has shown promising efficacy in refractory chronic lymphocytic leukemia [14]. Phase I trials of flavopiridol in children have revealed that its toxicity profile, pharmacokinetics, and maximum tolerable dose were similar to that in adults, indicating that using flavopiridol in RT patients, a largely pediatric population, is feasible [15].

The effects of flavopiridol on cancer cells are varied and cell type dependent. In many cell lines flavopiridol leads to G**_1 _**arrest due to down-modulation of cyclin D1 and inhibition of its pathway by various mechanisms [16-24]. In other cells, flavopiridol induces G**_2 _**arrest, in part due to its potent ability to inhibit cdk 7, 8 and 9 activities [25]. Flavopiridol also inhibits transcription of Mdm-2 resulting in the accumulation of its proteolytic target, p53, which triggers p21^Waf1 ^up-regulation, cyclin B1 down-regulation, and ultimately G_2 _arrest [26]. Flavopiridol can induce apoptosis at nanomolar concentrations and its pro-apoptotic action is either caspase-dependent or -independent [25]. Flavopiridol can trigger apoptosis by activation of caspases 2, 3 and 8 [27] or by activation of apoptosis inducing factor (AIF) via its release from the mitochondria [28]. At this point, the mechanism of action of flavopiridol in RT cells is not completely understood.

Since flavopiridol can be toxic at high doses, recent studies have focused on combining low concentrations of flavopiridol with other anti-neoplastic agents [29,30]. In this report, we tested the combination of flavopiridol with 4OH-Tam to determine its ability to increase therapeutic efficacy against RT cells. 4OH-Tam inhibits tumor cell growth in part by deregulating cyclins and cdks. Breast cancer cells over-expressing cyclin D1 are resistant to 4OH-Tam and the level of c*yclin D1 *is negatively correlated to responsiveness to 4OH-Tam [31-34]. 4OH-Tam suppresses the growth of estrogen-receptor positive tumors by down-modulating cyclin D1 [35]. Furthermore, treatment of tumor cell lines with 10μM 4OH-Tam induces the expression of p21^Waf1 ^and p27^Kip1^, which are known to block the effects of cyclin D1 [36]. Previous studies have demonstrated that 4OH-Tam is effective in inducing cytotoxic effects in RT cells [37]. In this study it was demonstrated that the expression of ERα receptor in RT cells is variable and that the cytotoxic effects of 4OH-Tam are independent of ERα expression [37]. Since the efficacy of flavopiridol in xenograft RTs was correlated with down-modulation of cyclin D1 and up-regulation of p21^Waf1^[13], we considered combining 4OH-Tam with flavopiridol to enhance its therapeutic efficacy in RT cells.

We report here that the combination of flavopiridol with 4OH-Tam potently inhibited the survival of RT cells. A low concentration of flavopiridol (100 nM) induced G_2 _arrest in RT cells in a p53-dependent manner and resulted in a moderate amount of apoptosis. Addition of 4OH-Tam significantly increased flavopiridol-mediated apoptosis. Down-modulation of p53 did not affect 4OH-Tam-induced cytotoxicity, but significantly enhanced flavopiridol-mediated apoptosis. Furthermore, we found that the increased cytotoxic effects of flavopiridol and 4OH-Tam correlated with augmentation of caspase 2 and 3 activities and that these effects were independent of p53. These studies indicate that the combination of flavopiridol and 4OH-Tam can be effective in potently inhibiting RT cell growth and is potentially a novel therapeutic strategy against RTs.

## Methods

### Cell culture and drug treatment

MON [6], G401 (American Type Culture Collection), and A204 cells (American Type Culture Collection) were maintained in RPMI supplemented with 10% fetal bovine serum, 50 U/ml penicillin, 50 μg/ml streptomycin, and 2 mM L-glutamine. For drug studies, the cells were transferred one day before drug treatment to RPMI containing 10% charcoal and dextran treated fetal bovine serum (HyClone, Cat. SH30068.03). Flavopiridol was obtained from the CTEP program at NCI (courtesy of Dr. Colevas). 4OH-Tam and pan-caspase inhibitor z-VAD-FMK were purchased from SIGMA (Catalogue #H7904) and Promega (Catalogue #G7232) respectively.

### Cell Survival analysis

8,000 cells/well (MON and A204) or 6,500 cells/well (G401) were plated in 96-well plates and treated with different concentrations/combinations of drugs, using the epMotion 5070 automated liquid handler system (Eppendorf). The CellTiter 96 AQueous One Solution Cell Proliferation Assay was used to determine cell survival (Promega, Catalogue #G3580). Data elaboration of cell survival, IC_50_, and drug combination effects were performed according to previously described methods [11].

### FACS Analysis

Propidium iodide staining and FACS analysis were performed as described previously [9]. Annexin staining was performed using Annexin V-FITCH Apoptosis Detection Kit I (BD Pharmingen Catalogue #556547) according to the manufacturer's instructions. Data was elaborated using CellQuest Pro program (BD Pharmingen).

### Statistical analysis

Data was analyzed using GraphPad Prism by applying the ANOVA test or t-test.

### Immunoblot analysis

Immunoblot analysis was carried out as described previously with minor modifications [9]. Dry milk was used as a blocking agent for the following antibodies: p53 (Santa Cruz Catalogue #sc-126), cyclin A (Santa Cruz Catalogue #sc-239), cyclin B1 (Santa Cruz Catalogue #sc-752), cyclin E (LabVision Catalogue #MS-870-P), cyclin D1 (Lab Vision Catalogue #RB-010-P), E2F-1 (Santa Cruz Catalogue #sc-251), GAPDH (Chemicon Catalogue #MAB374) and Rb (Santa Cruz Catalogue #sc-102); and bovine serum albumin for the following antibodies: cdk 2 (Santa Cruz Catalogue #sc-163), cdk 4 (Santa Cruz Catalogue #sc-260), cdk 6 (Santa Cruz Catalogue #sc-7961) and p21 (Calbiochem Catalogue #OP64). Chemiluminescence detection was achieved using SuperSignal West Pico Chemiluminescence Substrate (Pierce Catalogue #34080).

### RNA interference

MON cells were plated at 300,000 cells/well in 6-well plates one day before transfection. Cells were transfected using DharmaFECT siRNA transfection reagent (Dharmacon Catalogue #T-200(01-07)-01) according to the manufacturer's instruction and using previously published p53 siRNA (Qiagen Catalogue #024849) and control (Cy3-Luciferase GL2 Duplex, Dharmacon Catalogue #D-001110-01-05). One day post-transfection, cells were split into 50,000 cells/well using medium containing 10% charcoal and dextran treated fetal bovine serum, for drug treatment the following day.

### Immunocytochemistry

MON cells were grown on glass coverslips and treated with drugs. Cells were fixed with 4% paraformaldehyde-PBS solution for 10 minutes, and permeabilized with 0.1% triton-PBS solution for 10 minutes. Cover-slips were treated for 1 hour at room temperature with 1:250 diluted αp21 antibody (Calbiochem Catalogue #OP64). Staining was detected using the Vectastain ABC kit (Vector Laboratories Catalogue #PK-6102) according to manufacturer's instruction. Peroxidase staining was developed using the DAB enhanced liquid substrate system (SIGMA Catalogue #D3939-1SET). The percentage of cells with nuclear expression of p21 was quantified by counting 250 to 300 individual cells, noting whether or not their nuclei showed positive staining above the background. The background staining was defined as an intensity of staining at or below the intensity of the negative control (i.e. any background staining that occurred in the absence of a p21-specific antibody).

### Caspase-profiling assays

Caspase 3/7 Assay was performed using the Caspase-Glo 3/7 Assay kit (Promega Catalogue #G8093). Caspase activity profiling assays were performed using ApoAlert Caspase Profiling Plate (Clontech Catalogue #630225), according to the manufactures' instruction with minor modification, using 50 μg of protein for each well of the assay.

## Results and Discussion

### Combination treatment with flavopiridol and 4OH-Tam inhibits the growth of RT cells

**Figure 1:**
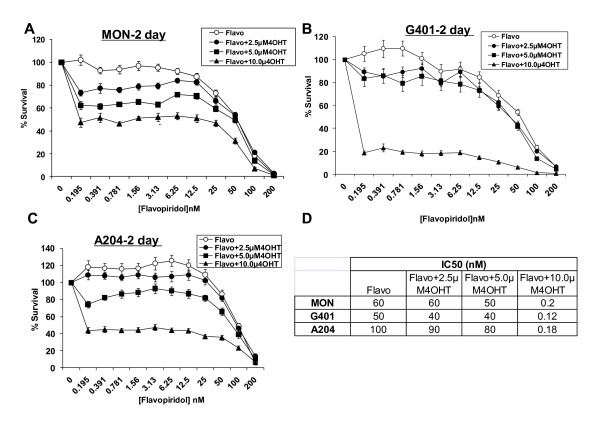
**Inhibition of rhabdoid tumor growth by flavopiridol and 4OH-Tam**. **A-C**. Survival curves of MON (A), G401 (B), and A204 (C) cells treated with flavopiridol in the presence and absence of 4OH-Tam for two days. **D**. Table of IC50 values as approximated from A-C. Flavo = flavopiridol; 4OHT = 4OH-Tam.

**Figure 2:**
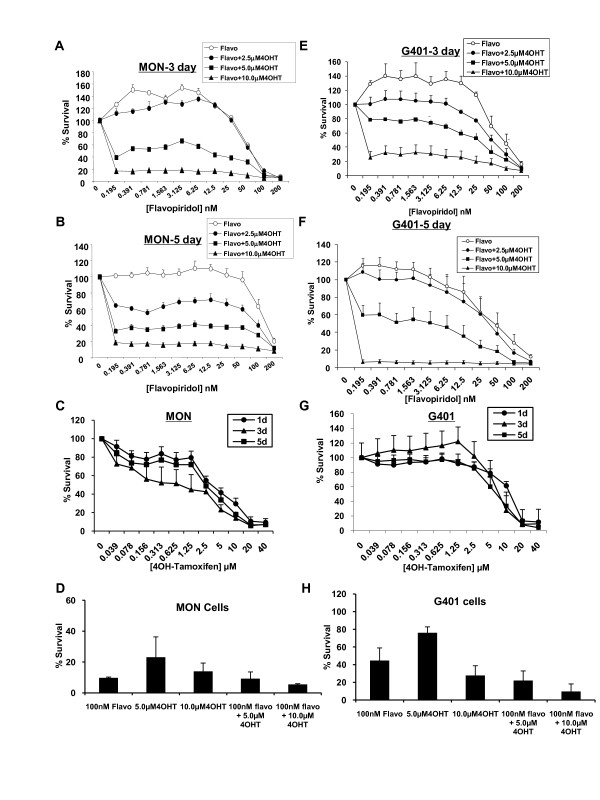
**Inhibition of rhabdoid tumor growth by flavopiridol and 4OH-Tam after prolonged treatment**. **A, B, E, and F**. Survival curves of MON (A-B)) or G401 (E-F) cells treated with flavopiridol in the presence and absence of 4OH-Tam for three (A and E) and five (B and F) days. **C and G**. Survival curves of MON (C) or G401 (G) cells treated with 4OH-Tam for one, three, and five days. **D and H**. Bar graph representing the decreases in survival of MON (D) or G401 (H) cells after treatment with specific concentrations of drugs for three days. (Flavo = flavopiridol; 4OHT = 4OH-Tam).

To determine the effect of combining flavopiridol with 4OH-Tam, we treated MON, G401, and A204 RT cells with increasing concentrations of flavopiridol (0 to 200 nM) in combination with 2.5, 5.0, or 10.0 μM 4OH-Tam for two days. Flavopiridol inhibited RT cell growth with IC_50 _values of ~50-100 nM, with MON cells showing an intermediate level of sensitivity compared to G401 and A204 cells (Figure 1A-D). Exposure of all cells to flavopiridol and 4OH-Tam in combination resulted in a dramatic reduction in IC_50 _values, with 10 μM 4OH-Tam reducing the IC_50_s to ~0.12-0.2 nM (Figure 1A-D). Furthermore, increasing the time of exposure to the combination of drugs enhanced the inhibitory effects in both MON and G401 cells (compare Figures 1A and 1B with Figure 2A, B, E, and 2F). Bar graphs derived from the data in Figure 2A and 2E are given to show the decreases in cell survival induced by the specific concentrations of the drugs after three days of exposure (Figure 2D and 2H). We also tested the effect of 4OH-Tam alone and found that 4OH-Tam decreased cell survival significantly (Figure 2C and 2G). However, increasing time of exposure to 4OH-Tam alone from one to three or five days, did not result in further decreases of cell survival (Figure 2C and 2G). Thus, while the flavopiridol and 4OH-Tam combination resulted in a time dependent decrease of survival, the effect of 4OH-Tam alone was independent of time of exposure. Notably, by day five of treatment, combinations of very low concentrations of flavopiridol (~ 0.2 nM) and 10 μM 4OH-Tam decreased cell survival by 80% in MON and 99% in G401 cells (Figure 2B and 2F). These results indicated that treatment with low concentrations of flavopiridol in combination with 4OH-Tam for prolonged periods potently inhibits RT cell growth.

### Induction of apoptosis by flavopiridol and 4OH-Tam

**Figure 3:**
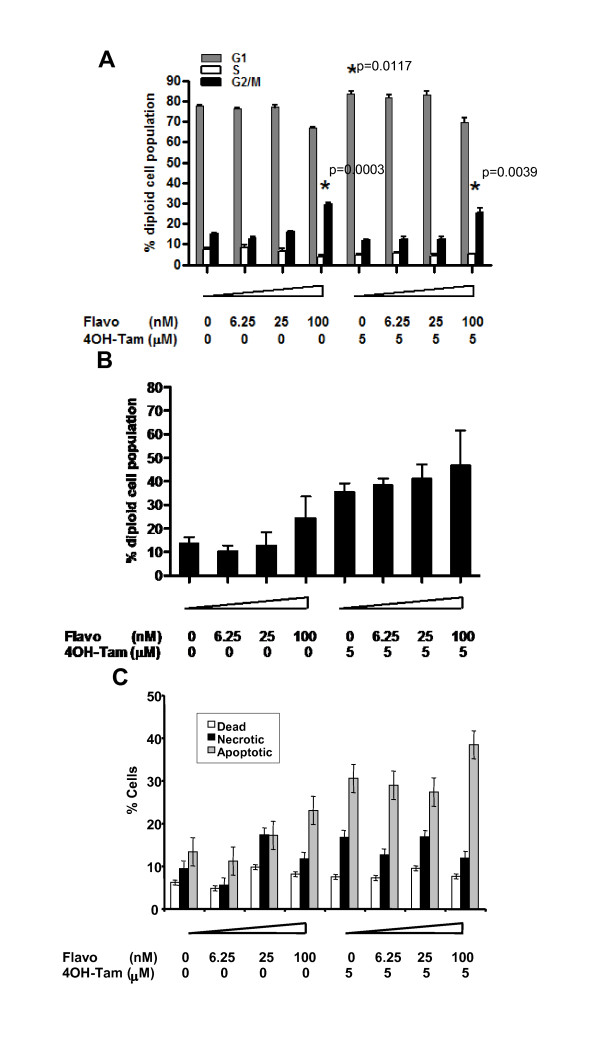
**Induction of cell cycle arrest and apoptosis by flavopiridol and 4OH-Tam**: **A**. Diploid cell cycle profile determined by FACS analysis of MON cells treated with flavopiridol and 4OH-Tam for two days; **B**. Percentage of MON cells at sub-G**_1 _**when exposed for two days to flavopiridol and 4OH-Tam **C**. Percentage of apoptotic (annexin positive), necrotic (PI positive) and dead (both annexin and PI positive) MON cells when exposed for two days to flavopiridol and 4OH-Tam.

To determine the mechanism of growth inhibition by these drugs, we studied MON cells which showed intermediate responses to the combination of drugs with an intermediate level of 4OH-Tam (5 μM) and duration of treatment. MON cells were exposed to combinations of flavopiridol and 5 μM 4OH-Tam for two days and subjected to cell cycle analysis. Treatment of cells with 5 μM 4OH-Tam alone resulted in G_1 _arrest (*p *= 0.0117, Figure 3A). Our previous results indicated that flavopiridol at 200-400 nM induces G_1 _arrest [13]. However, treatment with 100 nM flavopiridol induced G_2 _arrest in the presence or absence of 4OH-Tam (*p *= 0.0039 and *p *= 0.0003, respectively, Figure 3A) indicating that the stage of cell cycle arrest induced is dose-dependent. These results also indicate that G_2 _arrest induced by flavopiridol is dominant over 4OH-Tam-induced G_1 _arrest. Analysis of the sub-G_1 _fraction of cells after single or combination treatment indicated that the percentage of sub-G_1 _cells increased in a dose-dependent manner upon treatment with flavopiridol, which was further enhanced by combination with 4OH-Tam (Figure 3B). Very high levels of cell death are induced by these treatments and this likely plays the major role, compared to induction of cell cycle arrest, in inducing cytotoxicity in response to treatment with these drugs. To determine if cell death was due to apoptosis, the cells were stained for annexin (indicative of apoptosis) and propidium iodide (indicative of necrosis) and the percentage of cells positive for either or both stains was determined by FACS (Figure 3C). While the percentage of necrotic or dead cells remained largely unchanged, there was a dose-dependent increase in annexin-positive cells demonstrating that combination treatment with flavopiridol and 4OH-Tam induces significant levels of apoptosis.

### Induction of apoptosis by flavopiridol and 4OH-Tam is caspase-dependent

**Figure 4:**
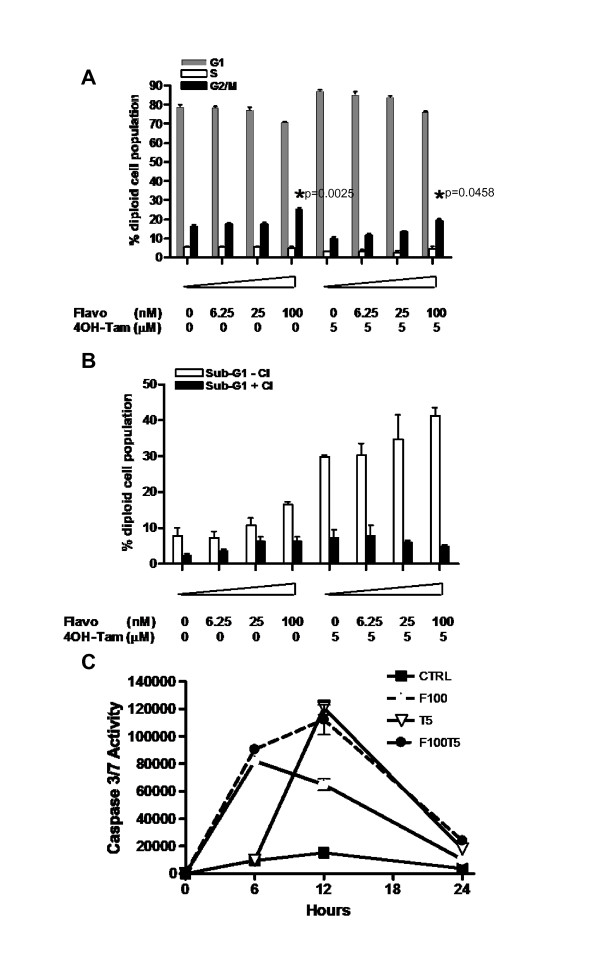
**Effect of caspases on flavopiridol- and 4OH-Tam-induced cell cycle arrest and apoptosis**: **A**. Diploid cell cycle profile determined by FACS analysis of MON cells treated with Flavopiridol and 4OH-Tam for two days in the presence of pan-caspase inhibitor Z-VAD-FMC (Compare to Figure 2A); **B**. Percentage of MON cells at sub-G_1 _when exposed for two days to flavopiridol and 4OH-Tam, in the absence and presence of pan-caspase inhibitor Z-VAD-FMC (CI). **C**. Kinetics of induction of caspase 3/7 activity in MON cells by flavopiridol and 4OH-Tam. The diagram illustrates the fold increase in caspase 3/7 activity at various time points after treatment. F100 = 100 nM flavopiridol; T5 = 5 μM 4OH-Tam.

Since it is known that flavopiridol can induce apoptosis by caspase-dependent or caspase-independent mechanisms, we investigated the effect of a caspase inhibitor on drug-induced cytotoxicity. MON cells were exposed for two days to flavopiridol and 4OH-Tam in the presence or absence of the pan-caspase inhibitor, Z-VAD-FMK, and FACS analysis was carried out to assess the effects on cell cycle and apoptosis. The results indicated that drug-induced cell cycle arrest was unaffected (*p *= 0.0458 and *p *= 0.0025 for G1-arrest induced by 100 nM flavopiridol with and without 4OH-Tam respectively) (Figure 4A), but induction of apoptosis by flavopiridol with or without 4OH-Tam was significantly inhibited by Z-VAD-FMK (*p *< 0.0001, Figure 4B). This established that cell death induced by flavopiridol, 4OH-Tam, or their combination is mediated by caspase-dependent apoptosis in RT cells. We further investigated the kinetics of induction of caspase 3 and 7 activities upon drug treatment. The results indicated that, while flavopiridol induced peak caspase 3 and 7 activities at six hours, 4OH-Tam exhibited delayed kinetics with peak induction at 12 hours. Interestingly, combination of 4OH-Tam and flavopiridol elevated caspase 3 and 7 activities at both 6 and 12 hours (Figure 4C). These results suggest that combination of flavopiridol and 4OH-Tam could increase apoptosis by inducing and prolonging caspase 3/7 activities.

### Mechanism of G_2 _arrest mediated by flavopiridol and 4OH-Tam

**Figure 5:**
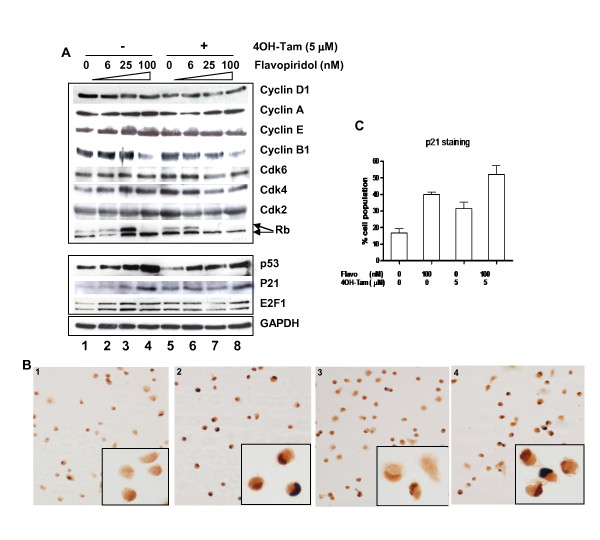
**Effect of flavopiridol and 4OH-Tam on expression of cell cycle regulatory proteins**: **A**. Immunoblots of various cell cycle proteins in the presence and absence of flavopiridol and 4OH-Tam, after two days of treatment. **B**. αp21 immunocytochemistry of MON cells treated with: no drug (panel 1), 100 nM flavopiridol (panel 2), 5 μM 4OH-Tam (panel 3), or both 100 nM flavopiridol and 5 μM 4OH-Tam (panel 4). Note the strong nuclear staining in the drug treated cells (panels 2-4). **C**. Quantitation of MON cells with nuclear p21 using α-p21 immunocytochemistry after treatment with no drug, 100 nM flavopiridol, 5μM 4OH-Tam, or 100 nM flavopiridol and 5μM 4OH-Tam. Average values represent counting of more than 200 cells in four random fields for each treatment group.

To determine the mechanism of cell cycle arrest induced by flavopiridol and 4OH-Tam, we examined the expression levels of several cell cycle regulatory and tumor suppressor proteins in the presence and absence of drugs. 100 nM flavopiridol induced p53 and p21^Waf-1 ^and repressed cyclin B1, consistent with the observed G_2 _arrest (Figure 5A). We found that the majority of cyclins and cdks, including cyclins D1, A and E, and cdks 2, 4 and 6 were unaffected by concentrations of flavopiridol ≤ 100 nM (Figure 5A). Interestingly, the presence of 4OH-Tam did not significantly alter the effects of flavopiridol on cyclin and cdk expression, but further decreased the level of pRb protein and phosphorylation (Figure 5A compare lanes 4 and 8). We also observed differential effects on pRb phosphorylation. Concentrations of flavopiridol ≤ 25 nM increased the level of phosphorylated pRb (Figure 5A lanes 2 and 3), but 100 nM dramatically decreased the phosphorylated form of pRb (Figure 5A lane 4). Addition of 4OH-Tam eliminated the flavopiridiol-induced phosphorylation of pRb at the 25 nM concentration (Figure 5A lane 7). However, 4OH-Tam had no effect on 100 nM flavopiridol-induced inhibition of pRB phosphorylation, consistent with the observed dominant effect of 100 nM flavopiridol in inducing G_2 _arrest.

### Flavopiridol induced p21^Waf-1 ^expression in RT cells

Our previous analysis indicated that flavopiridol efficacy in mouse xenografts correlated to both down-modulation of cyclin D1 and up-regulation of p21^Waf-1 ^[13]. Therefore, we examined p21^Waf-1 ^levels upon flavopiridol treatment with and without 4OH-Tam. We found that p21^Waf-1 ^was up-regulated in a dose-dependent manner upon treatment with flavopiridol (Figure 5A, lanes 1-4), but 4OH-Tam alone only modestly up-regulated p21^Waf-1^(Figure 5A, compare lanes 1 and 5). We also investigated the accumulation of nuclear p21^Waf-1 ^upon drug treatment by immunocytochemical analysis. The results indicated that flavopiridol treatment led to increased nuclear p21^Waf-1^, which was further increased upon addition of 4OH-Tam (Figure 5B and 5C). These results were confirmed by quantifying the percentage of cells with nuclear p21^Waf-1 ^(Figure 5C) and, taken together, indicate that combination treatment results in both increased protein levels and increased nuclear localization of p21^Waf-1^.

### Abrogation of p53 inhibited cell cycle arrest but enhanced apoptosis induced by flavopiridol

**Figure 6:**
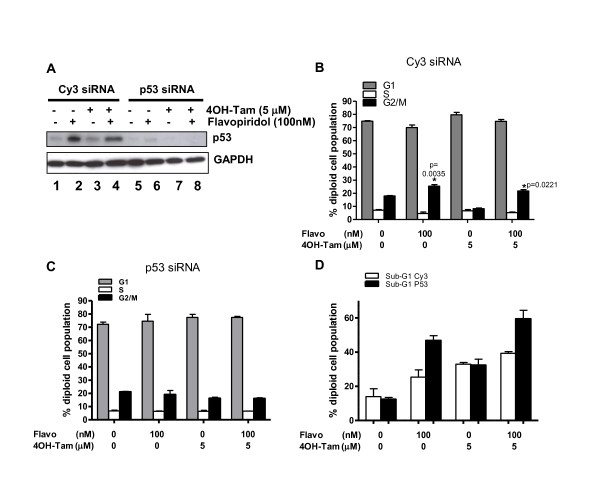
**Effect of p53 knock-down on flavopiridol- and 4OH-Tam-induced cell cycle arrest and apoptosis**: **A**. Immunoblot analysis to indicate siRNA mediated knock-down of p53 in MON cells treated with flavopiridol and 4OH-Tam for two days **B**. Percentage of diploid MON cells at various stages of cell cycle after treatment with control (Cy3) siRNA followed by treatment with flavopiridol and 4OH-Tam for two days. Note the lack of G**_2 _**arrest upon drug treatment of p53-knock-down cells. **C**. Percentage of diploid MON cells at various stages of cell cycle after p53 knockdown (using siRNA to p53) and treatment with flavopiridol and 4OH-Tam for two days. **D**. Percentage of MON cells in sub-G_1 _after transfection with control (Cy3) or p53 siRNAs and exposed to flavopiridol and 4OH-Tam for two days.

The above studies pointed to the involvement of p21^Waf-1 ^in flavopiridol or flavopiridol plus 4OH-Tam-mediated cytotoxicity. It has been established that p21^Waf-1 ^is a downstream effector of p53 [38]. Furthermore, tumorigenesis in *Ini1 *heterozygous mice is enhanced upon abrogation of p53 [39]. Since p53 is up regulated upon flavopiridol treatment, we further investigated its role. To determine whether p53 is necessary for flavopiridol-induced cell cycle arrest and/or apoptosis, RNA interference was used to down-modulate p53 expression. Transfection of RT cells with p53 siRNA slightly enhanced cell proliferation (data not shown). As expected, flavopiridol markedly increased p53 expression in cells transfected with control siRNA (Cy3), but not in cells transfected with p53 siRNA (Figure 6A, compare lanes 2 and 6). FACS analysis of cells transfected with control or p53 siRNA and treated with single or combination drugs for two days indicated that, while control siRNA did not affect drug-induced cell cycle arrest (p = 0.0221 and p = 0.0035 for G_1_-arrest induced by 100 nM flavopiridol with and without 4-OH-Tam respectively, Figure 6B), knock-down of p53 resulted in abrogation of flavopiridol-mediated G_2 _arrest both in the presence and absence of 4OH-Tam (p = 1.000 (not significant), Figure 6C). These results indicated that p53 is necessary for flavopiridol-mediated G_2 _arrest in RT cells.

Interestingly, knock-down of p53 demonstrated differential effects on flavopiridol and 4OH-Tam-mediated apoptosis in RT cells. Knock-down of p53 had no effect on 4OH-Tam-induced apoptosis indicating that 4OH-Tam-induced apoptosis is independent of p53 (Figure 6D). However, p53-knockdown enhanced flavopiridol-mediated apoptosis in the presence or absence of 4OH-Tam (Figure 6D). These results indicated that p53 is detrimental to flavopiridol-induced apoptosis in MON cells. Additionally, these results indicated that flavopiridol and 4OH-Tam induce apoptosis in RT cells by two different mechanisms; flavopiridol-mediated apoptosis being inhibited by p53 and 4OH-Tam-induced apoptosis being independent of p53.

### Role of caspases in flavopiridol and 4OH-Tam induced apoptosis

Expression of p53 has been associated with resistance to radiation-induced apoptosis in some cancers including gliomas and keratinomas [40,41]. Many RTs are resistant to chemotherapy and radiotherapy; however the mechanistic basis for this resistance is not clearly understood [42,43]. Based on our results we surmised that induction of p53 by flavopiridol is counter-productive to induction of apoptosis in RT cells. It has been reported that p53 is expressed in a majority of RTs and sequence analysis of mRNA does not show any abnormality in the p53 coding region [44,45]. Therefore, understanding the role of p53 in inhibiting drug-induced apoptosis might shed light on the mechanism of drug resistance exhibited by these tumors. Since flavopiridol increased p53 levels in RT cells, which was inversely correlated to induction of apoptosis, we explored the possibility that flavopiridol induced apoptosis through specific caspases. We profiled the kinetics of induction of caspase 2, 3, 8 and 9 activities in RT cells in the presence or absence of p53 and upon treatment with flavopiridol, 4OH-Tam, or their combination.

**Figure 7:**
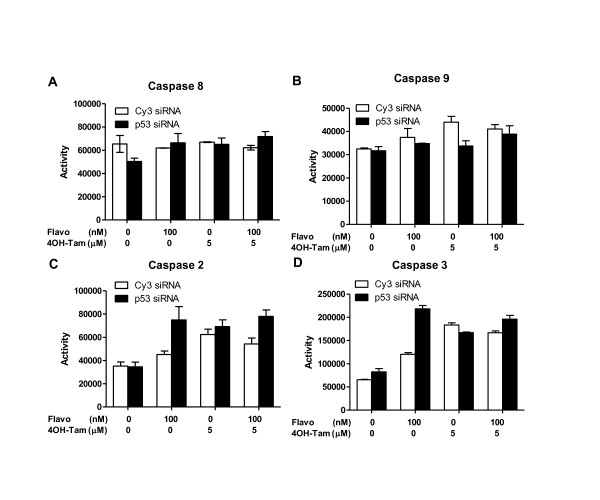
**Flavopiridol- and 4OH-Tam-induced caspase profiles in MON cells**. **A-D**. Induction of caspase 8 (A), 9 (B), 2 (C), and 3 (D) activities after 24 hrs of treatment with flavopiridol, 4OH-Tam, or the combination and treated with either control (Cy3) or p53 siRNA.

Analysis of the activation kinetics of caspases 2, 3, 8 and 9 indicated that neither flavopiridol nor 4OH-Tam induced caspase 8 and 9 activities in RT cells (Figure 7A and 7B and Additional File 1A-C). Induction of caspases 2 and 3 was affected by treatment with flavopiridol and flavopiridol with 4OH-Tam (*p *= 0.0022 for caspase 2 and *p*.0001 for caspase 3) and was correlated to p53 levels (*p *= 0.0074 for caspse 2 and *p *< 0.0001 for caspase 3) (Figure 7C and 7D). Flavopiridol increased caspase 2 and 3 activity two-fold within 6 hours and abrogation of p53 allowed flavopiridol to increase caspase 2 and 3 activity four-fold in the same time (Additional File 1D and 1G). Exposure of cells to flavopiridol with 4OH-Tam caused an overall increase in caspase 2 and 3 activity, with maximum induction observed at 12 hours (Figure 7C and Additional File 1F and 1I). Abrogation of p53 led to a further increase in caspase 2 and 3 activity after combination treatment, similar to that caused by flavopiridol alone (Figure 7C and Additional File 1F and 1I). 4OH-Tam alone caused only slight increases in caspase 2 and 3 activities in the presence or absence of p53 (Figure 7C and Additional File 1E and 1H).

To demonstrate that induction of caspase 2 and 3 activities were specific, we treated MON cells in the presence and absence of p53 and also in the presence and absence of specific caspase 2 or 3 inhibitors. Caspase 2 and 3 activities were determined at the 24 hr. time point in all of the treatment conditions. We found that treatment of cells with caspase 2 or caspase 3 inhibitors significantly reduced the induction of caspase 2 or 3 activities upon drug treatment respectively. These results indicated that flavopiridol and 4OH-Tam drug treatment selectively induces caspases 2 and 3 in MON cells, as inhibitors to these caspases eliminated these increases (Additional File 1J and 1K).

## Conclusions

Our report demonstrates, for the first time, that RT cell growth and survival is potently inhibited by combination treatment with clinically achievable concentrations of 4OH-Tam (2.5 or 5 μM) and flavopiridol (< 200 nM). Since high concentrations of flavopiridol may cause significant toxicities, and since only low concentrations may be achievable in areas such as the brain due to the blood brain barrier, our results provide a method to increase the efficacy of low concentrations of flavopiridol.

Flavopiridol and 4OH-Tam together induce a significant increase in RT cell death. Induction of cell death by flavopiridol and the combination of flavopiridol with 4OH-Tam is due to caspase-dependent apoptosis and caspase-profiling assays indicate that these treatments can potently induce caspases 2 and 3. In addition to inducing cell death, these treatments also induce cell cycle arrest. While our previous report indicated that 400 nM flavopiridol induces G_1 _arrest [13], this current report indicates that 100 nM flavopiridol is sufficient to cause G_2 _arrest indicating that flavopiridol potently induces cell cycle arrest in a dose-dependent manner. Flavopiridol-induced G_2 _arrest was correlated to down-regulation of cyclin B1 and up-regulation of p53 and p21^Waf-1^. On the contrary, 4OH-Tam inhibited p53 expression; however, this effect was nullified by addition of 100 nM flavopiridol, explaining the dominant effect of flavopiridol in mediating G_2 _arrest. These results suggest that the effect of flavopiridol in inducing p53 is upstream of the mechanism by which 4OH-Tam inhibits p53.

Interestingly, we found that p53 differentially regulated flavopiridol-mediated cell cycle arrest and apoptosis. RNA interference analysis of p53 indicated that while flavopiridol-mediated G_2 _arrest was dependent on p53, flavopiridol-mediated apoptosis (but not that mediated by 4OH-Tam) was countered by p53. This is an intriguing observation since there was a clear enhancement of apoptosis induced by flavopiridol when p53 was abrogated by RNA interference (Figure 6D). Previously it has been reported that lack of p53 enhances radio-sensitivity via activation of E2F-1 and induction of caspase 8 activity in glioma cells [40]. Furthermore, radio-sensitivity of keratinocytes was enhanced by abrogation of p53 and was mediated by down-regulation of anti-apoptotic proteins Mcl-1 and Bcl-_XL _[41]. To our knowledge, this is the first report which indicates that down-modulation of p53 also enhances drug-induced apoptosis. Our results indicate that the potency of flavopiridol can be enhanced if p53 can be inhibited by some means.

Most RTs express p53, though a percentage of RTs do show mutations within the p53 gene [44-46]. Some RT cell lines express p53 at high levels or with increased nuclear distribution, however, the p53 pathway has been tested and considered to be functionally intact [42,44]. The mutation status of p53 in MON RT cells has not been determined but the proper expression of p53 and responsiveness of p21^Waf1 ^to p53 levels leads us to believe that the p53 pathway is intact in these cells. However, other studies indicated that the pro-apoptotic pathway downstream of p53 may be dysfunctional in MON cells but the exact nature of this defect is unknown [43]. Although this defect in the pro-apoptotic pathway downstream of p53 could account for the observed effects reported here, our observation indicates that flavopiridol-induced apoptosis is inhibited by p53. More experiments are needed to delineate the exact role of the p53 pathway in flavopiridol-induced cytotoxicity in MON and other RT cells.

The relationship of flavopiridol and p53 in inducing apoptosis seems to be paradoxical in different cell lines and in different treatment approaches. Perhaps this is related to the paradoxical anti-apoptotic activities of p53 itself in various cancer cells [47]. The activities of p53 are cell type dependent and can be either pro-apoptotic and/or pro-survival. For example, in RT cells p53 is deleterious to flavopiridol-mediated apoptosis, but in other cancer cell lines flavopiridol-induced apoptosis is actually dependent on p53. An example is provided by Ambrosini *et al *who demonstrated that enhanced apoptosis induced by a combination therapy of flavopiridol with a G_1_-arrest-inducing agent (namely SN-38) was dependent on p53 [48]. In this study the combination of flavopiridol and SN-38 was tested on isogenic pairs of cells differing only in p53. They found that the enhanced apoptosis by combination of flavopiridol and SN-38 was observed only in p53^+/+ ^cells. Similar to SN-38, 4OH-Tam also induces G_1 _arrest in our system, however; the effects of combining flavopiridol with 4OH-Tam obtained in RT cells are different in terms of inducing apoptosis. While flavopiridol-mediated G_2 _arrest was dependent on p53, flavopiridol-induced apoptosis was abrogated by p53 (Figure 6C and 6D). On the contrary, p53 had no effect on 4OH-Tam-mediated apoptosis. Because of these results, we believe that p53 has compromising effects on flavopiridol-induced apoptosis in RT cells, similar to the effect of p53 in protecting cells from radiation induced apoptosis as discussed above.

Our studies involving p53 knock-down indicate that stimulation of p53 by flavopiridol limits its ability to induce apoptosis in RT cells. Thus, it is possible that 4OH-Tam increases the effects of flavopiridol because it down-modulates p53 in addition to inducing apoptosis by p53-independent mechanisms. This new understanding of p53's role in drug-induced apoptosis in RT cells might shed light on the mechanism of resistance to therapies exhibited by these tumors. Also, evaluation of p53 levels induced by flavopiridol and other treatments may be necessary to implement effective treatment strategies for RTs.

Flavopiridol induces apoptosis by additional p53-independent mechanisms. It is able to block RNA polymerase II phosphorylation by inhibiting cdk 9, thereby blocking transcriptional elongation. This activity, as well as flavopiridol's ability to reduce antiapoptotic protein MCL-1, has been implicated in the induction of apoptosis in multiple myeloma cell lines [49]. Additionally, induction of the mitochondrial permeability transition by flavopiridol has been correlated with induction of apoptosis in chronic lymphocytic leukemia cells [50]. These functions of flavopiridol may also contribute to the apoptosis occurring in RT cells in a p53-independent manner.

RTs are notoriously resistant to therapeutic interventions [3]. Potent chemotherapy in combination with surgery and radiotherapy have proven futile in increasing survival rates and only a handful of RT survivors have been reported [2]. Therefore, efforts to develop molecularly targeted therapies are needed. Based on the molecular understanding of RTs, it is known that INI1/hSNF5 mediates tumor suppression in part by targeting cyclins and cdks [7-9]. Furthermore, cyclin D1 is up-regulated in, and necessary for, rhabdoid tumorigenesis [9,12]. Thus, it appears that therapeutically inhibiting the cyclin/cdk pathway is a novel, targeted treatment strategy for RTs. Our report suggests that combination of flavopiridol and 4OH-Tam could be used as a novel combination therapy for RTs. Furthermore, this combination could be effective in inducing apoptosis in other tumor models, especially those lacking p53.

At this point, the concentrations of 4OH-Tam required to increase the effects of flavopiridol appear to be high. Nevertheless, in the pediatric population, where RTs most often occur, it has been reported that high doses of tamoxifen (100 mg/m^2 ^twice a day) can be administered with minimal toxicity [51]. Furthermore, in cases where sustained high concentrations (≥ 10 uM) of 4OH-Tam may not be attained, alternative formulations, such as a liposomal formulation, of both tamoxifen and 4OH-Tam have been used that would result in the delivery of high concentrations directly to the tumor [52]. Therefore, further preclinical and clinical studies to test the efficacy of these drugs in children may lead to the development of definitive therapeutic strategies against RTs that may improve prognosis.

## Competing interests

The authors declare that they have no competing interests.

## Authors' contributions

VC carried out FACS analysis, most of the immunoblot analysis, caspase profiling, p53 and p21 analysis, as well as participated in the design of the study, analyzed and interpreted the data, and participated in the initial preparation of the manuscript. MES participated in the design of the study, analyzed and interpreted part of the data, drafted the manuscript, carried out cell survival assays, and part of the immunoblot analysis. ZZ initiated the studies on the treatment of rhabdoid tumor cells with combination of flavopiridol and 4OH-Tamoxifen in the laboratory. DM participated in part of the survival assay, SM participated in the design of the study, provided guidance in the initial part of the study and preparation of the manuscript. GVK conceived of the study, designed the experiments, analyzed and interpreted the data, prepared the manuscript, and provided guidance for the entire study. All authors read and approved the final manuscript.

## Pre-publication history

The pre-publication history for this paper can be accessed here:

http://www.biomedcentral.com/1471-2407/10/634/prepub

## Supplementary Material

Additional File 1 **Flavopiridol- and 4OH-Tam-induced caspase profiles in MON cells**: **A-I**. Panels represent kinetics of induction of caspase activities in MON cells transfected with control (+Cy3) or p53 (+p53) siRNAs and then treated with 100 nM Flavopiridol (F100), 5 μM 4OH-Tam (T5), or both (F100T5) for two days. **J-K**. Graphic representation of caspase 2 and 3 activities induced by the drugs (as in A-I) in the presence of control (Cy3) or p53 siRNA (p53), at 24 hr. time point, in the presence or absence of specific caspase 2 and 3 inhibitors, respectively. The values illustrate the average of two independent experiments. Click here for file
