# Supplementary material for: Potent inhibition of rhabdoid tumor cells by combination of flavopiridol and 4OH-tamoxifen
Source: BMC Cancer. 2010 Nov 19;10:634. doi: 10.1186/1471-2407-10-634 (PMC2998513; doi:10.1186/1471-2407-10-634)
Supplement: Additional File 1 — Flavopiridol- and 4OH-Tam-induced caspase profiles in MON cells: A-I. Panels represent kinetics of induction of caspase activities in MON cells transfected with control (+Cy3) or p53 (+p53) siRNAs and then treated with 100 nM Flavopiridol (F100), 5 μM 4OH-Tam (T5), or both (F100T5) for two days. J-K. Graphic representation of caspase 2 and 3 activities induced by the drugs (as in A-I) in the presence of control (Cy3) or p53 siRNA (p53), at 24 hr. time point, in the presence or absence of specific caspase 2 and 3 inhibitors, respectively. The values illustrate the average of two independent experiments. [file 1471-2407-10-634-S1.PDF]

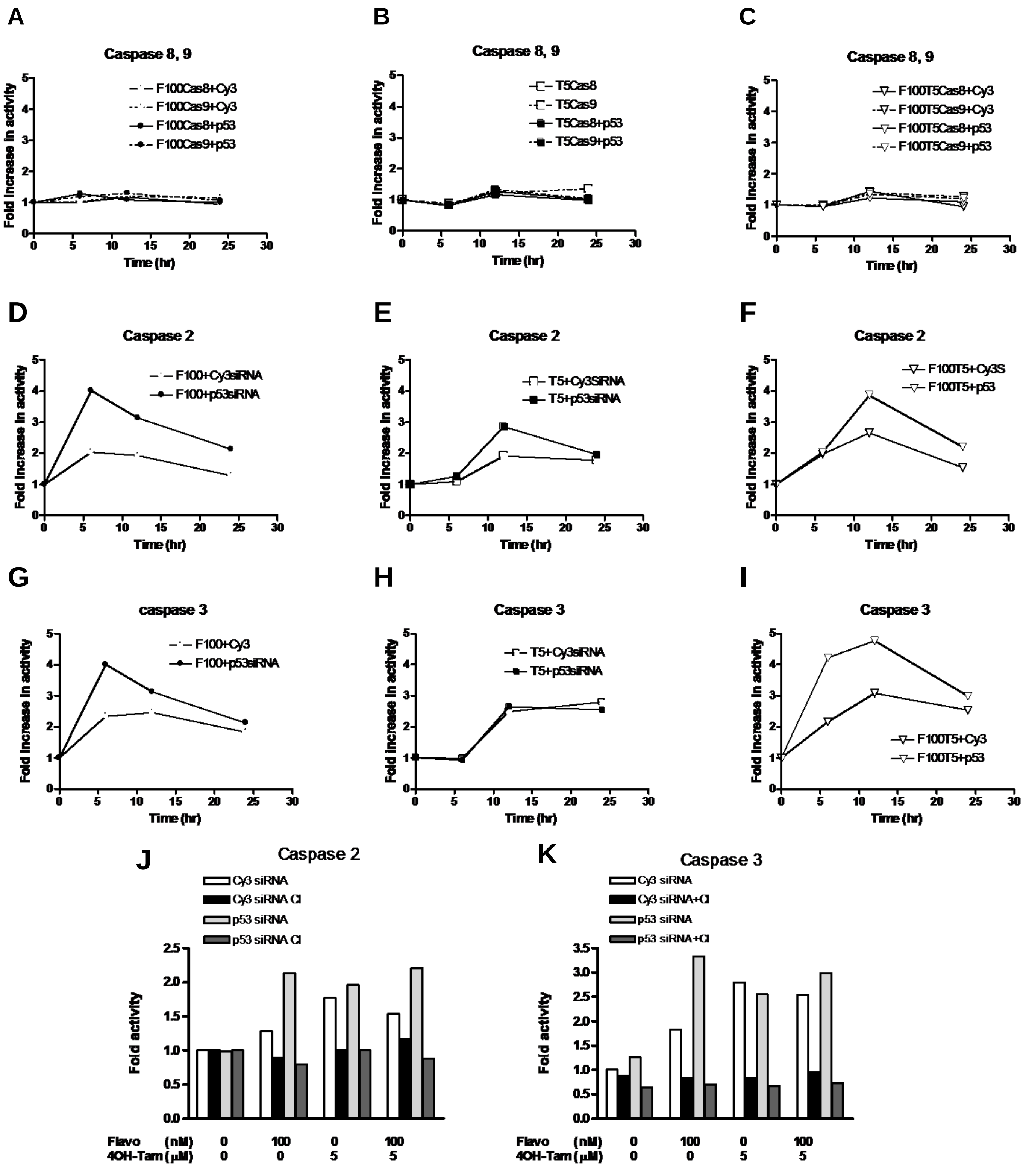
A
Caspase 8, 9
F100Cas8+Cy3
F100Cas9+Cy3
F100Cas8+p53
F100Cas9+p53
Fold Increase in activity
Time (hr)
B
Caspase 8, 9
T5Cas8
T5Cas9
T5Cas8+p53
T5Cas9+p53
C
Caspase 8, 9
F100T5Cas8+Cy3
F100T5Cas9+Cy3
F100T5Cas8+p53
F100T5Cas9+p53
D
Caspase 2
F100+Cy3siRNA
F100+p53siRNA
E
Caspase 2
T5+Cy3SiRNA
T5+p53siRNA
F
Caspase 2
F100T5+Cy3S
F100T5+p53
G
caspase 3
F100+Cy3
F100+p53siRNA
H
Caspase 3
T5+Cy3siRNA
T5+p53siRNA
I
Caspase 3
F100T5+Cy3
F100T5+p53
J
Caspase 2
Cy3 siRNA
Cy3 siRNA CI
p53 siRNA
p53 siRNA CI
Fold activity
Flavo (nM) 0 100 0 100
4OH-Tam (μM) 0 0 5 5
K
Caspase 3
Cy3 siRNA
Cy3 siRNA+CI
p53 siRNA
p53 siRNA+CI
Fold activity
Flavo (nM) 0 100 0 100
4OH-Tam (μM) 0 0 5 5
